# MNAzymes and gold nanoparticles as isothermal signal amplification strategy for visual detection of miRNA

**DOI:** 10.1007/s00604-023-05868-y

**Published:** 2023-07-17

**Authors:** Adrián Sánchez-Visedo, Borja Gallego-Martínez, Luis José Royo, Ana Soldado, Marta Valledor, Juan Carlos Campo, Francisco Javier Ferrero, José Manuel Costa-Fernández, María Teresa Fernández-Argüelles

**Affiliations:** 1 Department of Physical and Analytical Chemistry, University of Oviedo, Avenida Julian Clavería 8, 33006 Oviedo, Asturias Spain; 2 Health Research Institute of Asturias, ISPA, Avenida Hospital Universitario, s/n 33011 Oviedo, Asturias Spain; 3 Department of Functional Biology, Genetics, University of Oviedo, Avenida Julián Claveria, s/n 33006 Oviedo, Asturias Spain; 4 Department of Electrical, Electronic, Communications and Systems Engineering, University of Oviedo, Campus of Gijón, 33204 Gijón, Spain

**Keywords:** microRNA, MNAzymes, Signal amplification, AuNPs, Color detection

## Abstract

MicroRNAs (miRNAs) represent a class of small noncoding RNAs that are considered a novel emerging class of disease biomarkers in a variety of afflictions. Sensitive detection of miRNA is typically achieved using hybridization-based methods coupled with genetic amplification techniques. Although their sensitivity has improved, amplification techniques often present erroneous results due to their complexity. In addition, the use of these techniques is usually linked to the application of protein enzymes, the activity of which is dependent on the temperature and pH of the medium. To address these drawbacks, an alternative genetic enzyme for the highly sensitive detection of miRNAs is proposed in this work. Multicomponent nucleic acid enzymes (MNAzymes), coupled with the use of DNA-functionalized gold nanoparticles (AuNPs), were used in this study to develop an isothermal signal amplification strategy for visual genetic detection. miR146a, a biomarker of bovine mastitis present in milk, was selected as a model analyte. The developed methodology is easily carried out in 80 min at 50 °C, generating a low visual limit of detection of 250 pM based on the observation of a color change. The methodology was successfully applied to the detection of miR146a in raw cow milk samples.

**Graphical Abstract:**

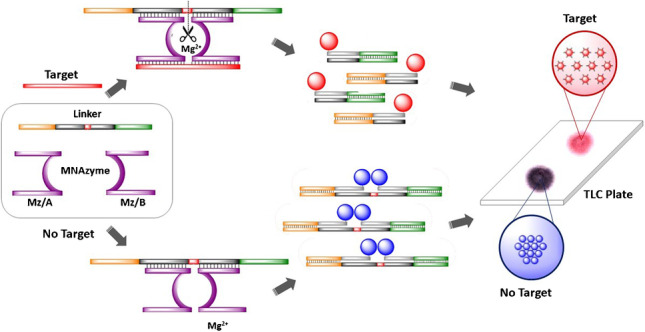

**Supplementary Information:**

The online version contains supplementary material available at 10.1007/s00604-023-05868-y.

## Introduction

MicroRNAs (miRNAs) are highly conserved single-stranded RNAs composed of a few nucleotides. This type of RNA sequence is involved in many different biological processes, such as protein expression and regulation, and is an emerging class of disease biomarkers. In this regard, several publications report a correlation between the presence of miRNAs and inflammatory diseases [1]. This is true for miR146a, as the presence of miR146a in dairy cattle is related to the immune response triggered by bovine mastitis, a condition that provides an indicator of the raw cow milk quality process and the hygienic conditions in farms [2]. Therefore, the development of a specific and sensitive method for detecting miR146a could potentially constitute a novel approach to the early diagnosis of bovine mastitis.

The detection of genetic targets through hybridization methods ensures excellent specificity for multiple bacterial infections [3], viruses [4], and tumoral cells [5]. However, sensitivity is often insufficient for detecting targets at clinically relevant levels. Thus, many genetic detection methods are based on amplifying a target sequence, such as real-time quantitative polymerase chain reaction (qPCR) [6]; alternatively, several recent isothermal amplification schemes can be used, including rolling circle amplification [7], hybridization chain reaction [8], and loop-mediated isothermal amplification [9]. However, amplification of the target sequence provides a high percentage of erroneous results due to poor primer design or contamination, causing the accumulation of false positives [10]. Other types of schemes described in the literature are based on direct target detection with further amplification of the signal using protein enzymes. Those methods lead to target detection with low detection limits, avoiding target amplification errors. In these works, direct detection of the target sequence is carried out through hybridization with complementary strands labeled with enzymes to provide the amplified signal [11]. Despite the advantages of these methods, they involve several inconveniences, as semiquantification (rather than absolute quantification) is achieved, accurate normalization is needed, and the sample preparation steps before amplification are time-consuming.

**Fig. 1:**
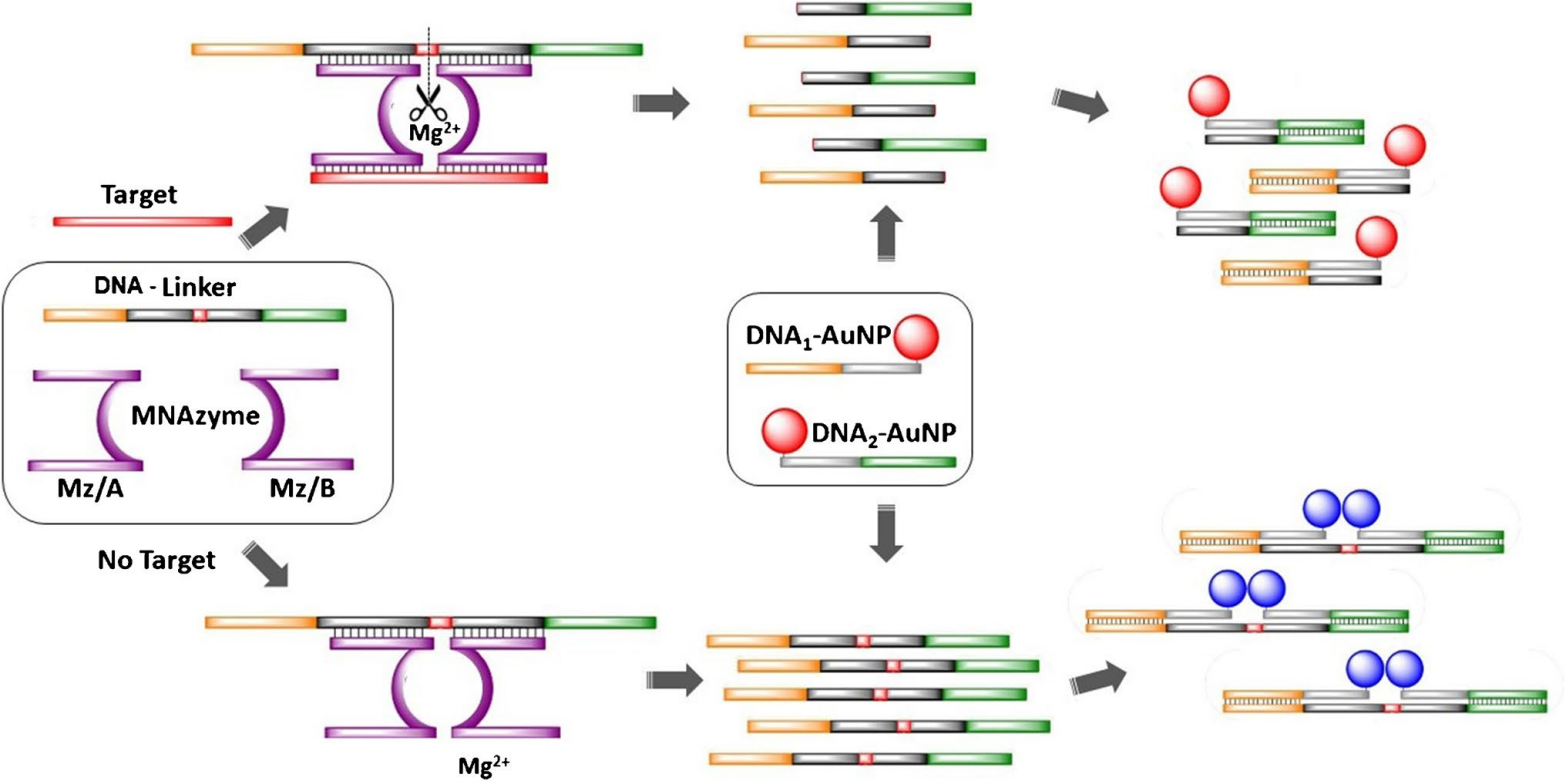
Scheme for the detection of a microRNA target

As an alternative, research employing genetic enzymes, also known as DNAzymes, uses nucleotides as a principal building component. This allows stringent assay conditions, including temperature and pH of the medium, that are not possible when using protein enzymes [12, 13]. Among the different types of DNAzymes that have been described in the literature, those containing a catalytic core with RNA cleavage activity have been widely studied [14–16]. A modification of this type of cleaving DNAzymes has led to a novel structure called multicomponent nucleic acid enzymes (MNAzymes) [17]. MNAzymes are derived from DNAzymes and are composed of the following parts: (i) a catalytic core, (ii) sensor arms that are complementary to the target sequence, and (iii) and substrate-binding arms complementary to another sequence (known as substrate or DNA-linker) that contains two RNA bases through which cleavage occurs (see Fig. 1). This cleavage only occurs in the presence of a target upon hybridization with the sensor arms of the MNAzyme; in the absence of the target, the substrate sequence remains intact, whereas, in the presence of the target, the substrate is broken into two shorter strands. Additionally, cleavage takes place in multiple turnovers because one sequence of the target can produce the cleavage of several substrate sequences. As a result, if the substrate is appropriately labeled on both ends, changes in the distance between the labels due to substrate cleavage can be exploited to design highly sensitive methodologies. Additionally, the method is extraordinarily flexible for genetic applications due to the lack of protein enzymes and the ability to easily modify the sensor and substrate arm sequences depending on the target.

Through modifying the surface of gold nanoparticles (AuNPs) with oligonucleotides, they can be used in many bioanalytical applications based on changes in the distance between AuNPs upon hybridization with the target. The detection involves variation in the surface plasmon resonance (SPR) absorption wavelength between aggregated and nonaggregated AuNPs. In this sense, according to previous works published in the literature [18], AuNPs can be easily combined with genetic hybridization techniques to develop rapid and cost-effective optical methodologies for miRNA detection [19, 20]. However, these methods may not be adequate to detect ultralow levels of the target due to their lack of sensitivity.

Taking advantage of the catalytic activity of the MNAzymes, a new methodology is proposed here to visually detect low concentrations of miR146a. To accomplish this, short DNA sequences that are partially complementary to a substrate sequence containing two RNA bases are used to modify AuNPs. These surface-modified AuNPs are then combined with MNAzymes that contain substrate-binding arms complementary to the substrate and sensor arms that are complementary to miR146a. When miR146a is present, it activates the MNAzyme, resulting in the cleavage of the substrate in multiple turnovers. The cleavage causes an increase in the distance between the AuNPs, which leads to a change in the color of the NPs to red. However, the substrate remains complete due to the absence of miR146a, so the aggregation of AuNPs takes place, and a change to a purple color can be observed. To simplify the readout of the assay, the aggregation state can be visualized using a C18 silica thin-layer chromatography (TLC) plate, in which the AuNPs are placed; thus, the color can be simply observed without instrumentation, and the tested samples can be stored in a permanent, stable, rapid, and cost-effective manner [21].

## Experimental

### Materials and reagents

Two thiolated DNA sequences have been used to modify the AuNPs surface, named as probe 1 (probe 1) for 5’-SH-AAA AAA AAA ACC TAT CGA CCA TGC T-3’ and probe 2 (probe 2) for 5’-TTT GCT GAG ATC GCG AAA AAA AAA A-SH-3’. The DNA DNA-linker sequence that is modified with two RNA bases, represented as rGrU, 5’-AGC ATG GTC GAT AGG TAA GGT TTC CTC rGrU CCC TGG GCA TAA ACG ACT CTA GCG C-3’; two strands from the MNAzyme (Mz/A) 5’-ACA ACC TAT GGA ACA ACG AGA GGA AAC CTT-3’ and (Mz/B) 5’-TGC CCA GGG AGG CTA GCT ATT CAG TTC TCA-3’; the target miRNA146a 5’-UGA GAA CUG AAU UCC AUA GGU UGU-3’ and the target with one or two base modifications (underlined and bold): 1 base modification (named 1U) 5’-UGA GAA CUG AAU **A**CC AUA GGU UGU-3’; 1 base modification: (named 1C) 5’-UGA GAA CUG AAU UC**U** AUA GGU UGU-3’; and two base modifications: (named 2GC) 5’-UGA GAA CU**C** AAU UC**U** AUA GGU UGU-3’ were obtained from Integrated DNA Technologies (Iowa, USA). Sodium citrate tribasic dihydrate (Na_3_C_6_H_5_O_7_ ∙ 2H_2_O), hydrogen tetrachloroaurate trihydrate (HAuCl_4_ ∙ 3H_2_O), Trizma® hydrochloride (NH_2_C(CH_2_OH)_3_ ∙ HCl), magnesium chloride hexahydrate (MgCl_2_ ∙ 6H_2_O), potassium chloride (KCl), and Tris-borate-EDTA buffer, 5× concentrate, and powder blend were purchased from Sigma-Aldrich (St. Louis, USA). Thiolated methoxy polyethylene glycol (mPEG-SH_1000_) was from Laysan Bio, Inc. (Huntsville, USA). For gel electrophoresis, SeaKem® LE Agarose was purchased from Cambrex Bio Science Rockland, Inc. (Rockland, USA). Thin-layer chromatography (TLC) aluminum sheets silica gel 60 F_254_ were obtained from Merck KGaA (Darmstadt, Germany). Milk samples were acquired from the Department of Animal Nutrition, Grassland and Forages, Regional Institute for Research and Agrofood Development (SERIDA, Asturias, Spain). RNA extraction was carried out using QIAzol Lysis Reagent from QIAGEN (USA), and mirVana microRNA isolation kit and phosphate-buffered saline (PBS) were from Thermo Fisher Scientific (USA).

### Synthesis of AuNPs and surface functionalization

AuNPs were synthesized according to a previously described method [22]. Characterization of morphology, size, and polydispersity index (PDI) of AuNPs was carried out initially using dynamic light scattering (DLS) Zetasizer Nano ZS (Malvern Panalytical, United Kingdom) to discard AuNPs obtained without low PDI and a JEOL-2000 EX-II Transmission Electron Microscope (TEM) (JEOL Ltd., Japan). The AuNPs were synthesized in our laboratory, with a diameter of 15 ± 1 nm (*n* = 500) with a PDI of 0.01, calculated from TEM images.

In order to perform the surface functionalization of the AuNPs, two different DNA sequences with a 5’-thiol-group (probe 1) and 3’-thiol-group (probe 2) were mixed with two colloidal dispersions of AuNPs to obtain DNA_1_-AuNP and DNA_2_-AuNP, respectively [18]. The colloidal stability of the bioconjugates was improved by adding thiolated methoxy polyethylene glycol (mPEG-SH_1000_) to the surface of the DNA-functionalized AuNPs. DNA_1_-AuNP and DNA_2_-AuNP were diluted with ultrapure water with 0.01% v/v Tween-20 to a final concentration of 5 nM, calculated using an extinction coefficient of 2.33 × 10^8^ M^−1^ cm^−1^ at λ = 521 nm for 15 nm AuNPs. Details of the experimental procedure are provided in the Supplementary information.

### MNAzyme amplification procedure

In a typical experiment, 4-μL standard solutions of the target at different concentrations (0–5000 pM) were mixed with 1 μL of MNAzyme 4 μM (Mz/A + Mz/B) solution and 2 μL of DNA-linker 400 nM in 3 μL of buffer assay (200 mM MgCl_2_ ∙ 6H_2_O, 0.1 M Trizma® HCl, and 0.5 M KCl, pH = 8.3) for 1 h at 50 °C. The DNA-linker strand (also named substrate) has two RNA bases that can be cleaved by an active MNAzyme. The MNAzyme becomes active only when the target hybridizes with the arms of both Mz/A and Mz/B in an Mg^2+^ medium at a certain pH [17]. Therefore, in the presence of the target, the MNAzyme turns into a catalytic structure capable of cleaving the DNA-linker strand through the RNA bases in two shorter pieces. Each MNAzyme catalyzes the cleavage of multiple DNA-linker strands, endowing a signal amplification (Fig. 1).

### Detection/AuNP aggregation assay

After the MNAzyme amplification step, 5 μL of DNA_1_-AuNP and 5 μL of DNA_2_-AuNP were added to the previous reaction mixture, containing the target, MNAzyme, and DNA-linker (either cleaved or intact as a function of the concentration of target) for 20 min at 50 °C. DNA_1_-AuNP and DNA_2_-AuNP partially hybridize with the DNA-linker strand. In the absence of the target, the DNA-linker remains intact, hybridizing at both ends with DNA_1_-AuNP and DNA_2_-AuNP, generating an aggregated network of AuNPs. This aggregation leads to a redshift of the SPR wavelength, changing the color of the NP dispersion from red to dark purple. Conversely, the presence of the target activates the MNAzyme, cleaving the DNA-linker to generate two smaller strands, each one of those hybridizing with DNA_1_-AuNP and DNA_2_-AuNP, respectively. Because the DNA-linker is broken into two pieces, the distance between DNA_1_-AuNP and DNA_2_-AuNP is high, so the DNA-modified AuNPs remain not aggregated after the assay, and the solution persists with the typical red color of well-dispersed AuNPs (see Fig. 1).

3 μL of the assay solution was then deposited onto a TLC plate, facilitating the visual differentiation between aggregated and not-aggregated AuNPs and allowing the naked-eye detection of the miRNA based on the aggregation estate of the NPs [22]. To obtain an image of the spots, a digital scanner (HP LaserJet Pro MFP M26nw) was employed to record all the TLC spots, avoiding the influence of ambient light when taking a picture of the spots. Furthermore, UV/Vis measurements of each sample were carried out to optimize assay conditions.

### Sample pre-treatment

Milk samples from dairy cattle were provided by the Regional Institute for Research and Agrofood Development of the Principality of Asturias (SERIDA). Following the procedure described in previous work, 50 mL of milk was centrifuged at 4000 g for 20 min at 4 °C to separate the different phases [18]. Fat and serum phases were discarded, and the pellet where the cells were found was used for further experiments. This pellet was washed several times by centrifugation cycles with PBS. Then, 2 mL of QIAzol reagent was added to lyse the cells. In the next step, known amounts of miRNA were added to each lysed sample, and RNA extraction was carried out using the mirVana kit, according to the manufacturer’s instructions. To estimate the miR146a concentration, a 70% yield for RNA extraction has been assumed [23].

## Results and discussion

### Principle behind the amplification strategy for miRNA detection

Figure 1 schematically represents the assay that was carried out in two stages, which were both performed at 50 °C. First, different concentrations of standard solutions of the target were mixed with the MNAzyme and DNA-linker solutions. The MNAzyme is composed of two oligomers (MzA, MzB) containing two sensor arms; these oligomers are partially complementary to the target, and their catalytic activity becomes active in an Mg^2+^ medium upon hybridization with the target [17]. MNAzymes also contain two substrate arms that are partially complementary to a substrate (i.e., DNA-linker), which is a DNA strand that contains two RNA bases that can be cleaved by an active MNAzyme. In the absence of the target, the MNAzyme is inactive, and the substrate remains intact. In contrast, in the presence of the target, the MNAzyme becomes active, forming a structure with a catalytic activity that hybridizes and cleaves the RNA bases of the substrate, generating two shorter strands. When one single target strand interacts with an MNAzyme, multiple substrate strands can be cleaved, which can be exploited to generate amplification of the signal. MNAzymes have already been described in the literature to detect longer DNA and RNA sequences. Nevertheless, they were not evaluated for the detection of short sequences, such as miRNAs. Hence, in the present work, the base composition and DNA-linker length were selected according to the literature, while the target arms of both partzymes were designed to be shorter and with a base composition complementary to the miRNA of interest. Thus, all sequences were hybridized under isothermal conditions due to the melting temperatures of the final assembly.

Next, the DNA surface-modified AuNPs were added to the medium. The surface of the AuNPs was previously modified with two different thiolated DNA oligomers (DNA_1_-AuNPs and DNA_2_-AuNPs). Each set of DNA-functionalized AuNPs is complementary to one end of the substrate. Hence, in the presence of the target, the substrate that is cleaved into two short strands in the first step hybridizes with each corresponding AuNP. Thus, the colloidal stability of the AuNPs is maintained, and the resulting dispersion shows a pink-red color. However, in the absence of the target, the MNAzyme is not active, so the substrate remains complete. Therefore, when the AuNPs are added, both sets of DNA_1_-AuNPs and DNA_2_-AuNPs hybridize with the two ends of the substrate, moving DNA_1_-AuNPs and DNA_2_-AuNPs in close proximity. This agglomeration of AuNPs can be observed due to the dark purple color of the mixture.

The color change of the colloidal dispersion due to the presence of the target can be instrumentally monitored by measuring the maximum absorption wavelength of the SPR peak [24] or by placing a 3-μL drop onto a TLC plate; as a result, two colors can be distinguished by the naked eye [24, 25].

### Assessment of DNA:AuNPs functionalization

The DNA:AuNP molar ratio employed in the assay was optimized following the method reported previously [18]. As well known, to achieve an appropriate functionalization of the AuNPs, their surface should contain the maximum possible number of DNA molecules. The DNA:AuNP stoichiometry depends on the diameter of the NPs, so several molar ratios were studied for AuNPs with a 15-nm diameter. These spherical AuNPs were selected because they are stable for long periods after bioconjugation and exhibit visible changes in color upon aggregation. The bioconjugates were prepared in a range of 0:1 up to 300:1 DNA:AuNPs, and an electrophoretic mobility assay was performed in 1% agarose for 40 min at 100 V. The study was carried out for both probe 1 and probe 2 because terminal functional groups of the commercial thiolated strands are slightly different depending on the position of the functionalization (3´ or 5´ end), even though the probes contain the same number of DNA bases.

As expected, studies revealed that when the surface of the AuNPs is not completely loaded with DNA (i.e., with low DNA:AuNP molar ratios), the bioconjugates migrate at a faster speed, and the bands generated are broader. However, from a certain DNA:AuNP ratio, the migration speed remains constant, and the bands are narrow regardless of whether higher molar ratios are employed, which indicates that the surface of the AuNPs is loaded with the maximum amount of DNA possible. As can be observed in Supplementary Fig. S1, the migration speed remained constant for 200:1 DNA:AuNPs molar ratios for both bioconjugates. Hence, this molar ratio was selected for further experiments.

The AuNP concentration (DNA_1_-AuNPs and DNA_2_-AuNPs) was also evaluated to ensure the most sensitive detection of the colorimetric assay was achieved. In this study, AuNP concentrations between 1 and 10 nM were evaluated, and 5 nM was selected for further experiments (detailed information in Supplementary Fig. S2).

### Selection of experimental parameters affecting the amplification

To achieve the best analytical performance in terms of sensitivity and reproducibility, different experimental parameters that might affect the response of the assay were evaluated, including DNA-linker, MNAzyme and MgCl_2_ concentrations, temperature, and the assay duration. The aforementioned experimental parameters affect both the hybridization process and the catalytic activity of the MNAzymes. Therefore, differences in the agglomeration state of the two sets of DNA-functionalized AuNPs in the presence of the target are also closely related to these variables.

The first experimental parameters studied were the time and temperature of the assay, which had a direct influence on the two stages of the detection test. In the first step, the catalytic activity of the MNAzyme should be maximized to achieve the highest possible amplification through the cleavage of the substrate. As previously determined, the rate of cleavage of RNA bases by the MNAzyme is influenced by many factors including temperature, length of the DNA-linker binding arms, constituents, and concentration of the buffer. A time-dependent catalytic signal was evaluated at different temperatures. For this purpose, changes in the SPR wavelength of the AuNPs were evaluated in the absence and presence of a high concentration of miR146a, at 40 °C, 45 °C, 50 °C, 55 °C, and 60 °C, from 20 to 80 min. Results obtained are detailed in Supplementary Fig, S3, where the maximum difference occurs when the cleavage step is performed for 60 min at 50 °C. In addition, the temperature of the cleavage step was also evaluated for the detection of different concentrations of miR146a (see Supplementary Section S.3.1. including the selection of the temperature of the assay and Supplementary Fig. S4). The results obtained showed that at 40 °C and 60 °C, no change in the aggregation state of the AuNPs was observed as the target concentration increased. However, when the temperature of the assay was set to 50 °C, a noticeable change in the aggregation of DNA:AuNPs was observed due to the activity of the MNAzmes. Therefore, an optimal catalytic temperature of 50 °C was selected for further experiments. These results are in agreement with previous publications based on the use of MNAzymes with similar features, such as the length of Mz substrate arms (between 10 and 22 bases) and MgCl_2_ concentration (50 mM) to ensure maximum cleavage of the substrate [17, 26]. In the second step of the assay, cross-linking occurs between the two sets of DNA-functionalized AuNPs, either with the whole DNA-linker or with the two short strands generated upon cleavage of the DNA-linker takes place. According to the melting temperatures of probe 1 (Tm: 54.1 °C) and probe 2 (Tm: 53.1 °C) provided by the manufacturer and considering that an isothermal assay is intended, an optimum temperature of 50 °C was also selected for the second step. The incubation time of both steps was also studied in order to allow the detection of the smallest possible concentration of miR146a within the shortest period of time. Results obtained from these studies are displayed and detailed in Supplementary Figs. S5 and S6 for the first and second steps, respectively. As shown in these results, incubation times of 60 min for the first step and 20 min for the second step were selected for further experiments because, in these conditions, the presence of 100 pM miR146a produces changes in the wavelength of the SPR peak.”

DNA-linker and MgCl_2_ concentrations are also critical parameters to achieve high sensitivity. If the concentration of DNA-linker or MgCl_2_ is too high, AuNPs will be aggregated, and the presence of a small concentration of target will not produce a noticeable change in the agglomeration state of AuNPs. On the other hand, if the concentration of the DNA-linker or MgCl_2_ is too low, no aggregation will occur between AuNPs in the absence of the target due to the high colloidal stability of the DNA-functionalized AuNPs; thus, no difference will be observed in the presence of the target. Hence, optimal DNA-linker and MgCl_2_ concentrations are the lowest possible concentration at which a suitable aggregation of AuNPs occurs without the target. This study was carried out without the target, ensuring that the aggregation of AuNPs was observed in the presence of inactive MNAzymes. Although a high salt concentration would improve the catalytic activity of the MNAzyme, AuNP aggregation would be induced even in the absence of a DNA-linker, which is a negative outcome. Therefore, the following MgCl_2_ concentrations were evaluated: 10 mM, 15 mM, 20 mM, and 30 mM. For each MgCl_2_ concentration study, different DNA-linker concentrations were evaluated, from 0 to 70 nM. The rest of the experimental parameters remained constant, as described in the “Experimental” section.

**Fig. 2:**
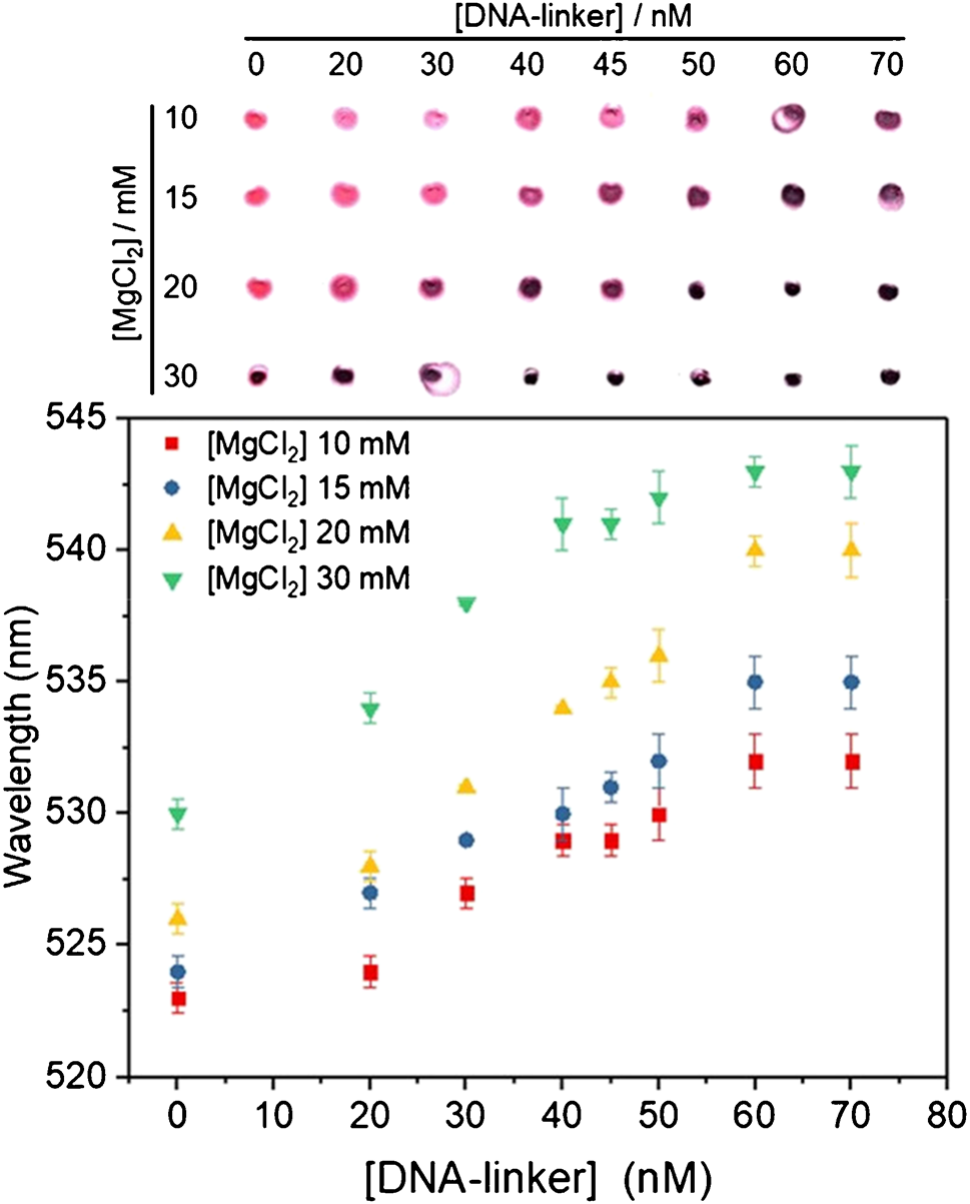
Optimizing the concentrations of [DNA-linker] and [MgCl_2_]. SPR wavelength and TLC spots of AuNPs at 10 mM, 15 mM, 20 mM, and 30 mM MgCl_2_ concentrations; and 0 nM, 20 nM, 30 nM, 40 nM, 45 nM, 50 nM, 60 nM, and 70 nM DNA-linker concentrations. All the experiments were performed in triplicate (*n* = 3)

Figure 2 summarizes the results from the optimization of the concentration of DNA-linker and Mg^2+^, in which a picture of the spots of the resulting mixture on a TLC plate shows the difference in the color observed and the shift of the wavelength of the SPR peak is represented against the DNA-linker concentration. These studies were performed in triplicate. As can be observed in the upper part of the figure, all AuNPs appear aggregated (dark blue/purple spots) at 30 nM MgCl_2_, even those without DNA-linker, indicating that this MgCl_2_ concentration is too high for the assay. However, if the concentration of MgCl_2_ is much lower (e.g., 10 mM), a high concentration of DNA-linker is needed to observe the aggregation of the AuNPs. Therefore, an optimum MgCl_2_ concentration could be between 15 and 20 mM. Using 20 mM MgCl_2_, a DNA-linker concentration of 40 nM provides a complete aggregation, and similar results can be observed for 15 mM MgCl_2_ and 50 nM DNA-linker, respectively. These results were also evaluated through spectrophotometric measurements by means of SPR wavelength shifts. As displayed in the lower part of the figure, 30 mM MgCl_2_ presents an SPR peak close to 530 nm in the absence of the DNA-linker, which is consistent with the observed color of the spot that indicates aggregation of AuNPs. For 10 mM and 15 mM MgCl_2_, the SPR wavelength is located around 530 nm and 533 nm, respectively, even if the DNA-linker concentration is above 60 nM; thus, aggregation is not complete. However, when evaluating the SPR wavelength shift at 20 mM MgCl_2_, noticeable aggregation is produced using a 40 nM DNA-linker, and an even higher degree of aggregation can be achieved with a 60–70 nM DNA-linker.

In brief, the highest slope of the curves and, therefore, maximum assay sensitivity was obtained at 20 mM MgCl_2_. In addition, although the highest wavelength shift was obtained for DNA-linker concentrations above 60 nM, more targets should be needed to cleave that concentration of DNA-linker, and 50 nM already provides very good visual aggregation. Therefore, 20 mM MgCl_2_ and 50 nM DNA-linker concentrations were selected for further experiments.

### Visual detection of miR146a with AuNPs

**Fig. 3:**
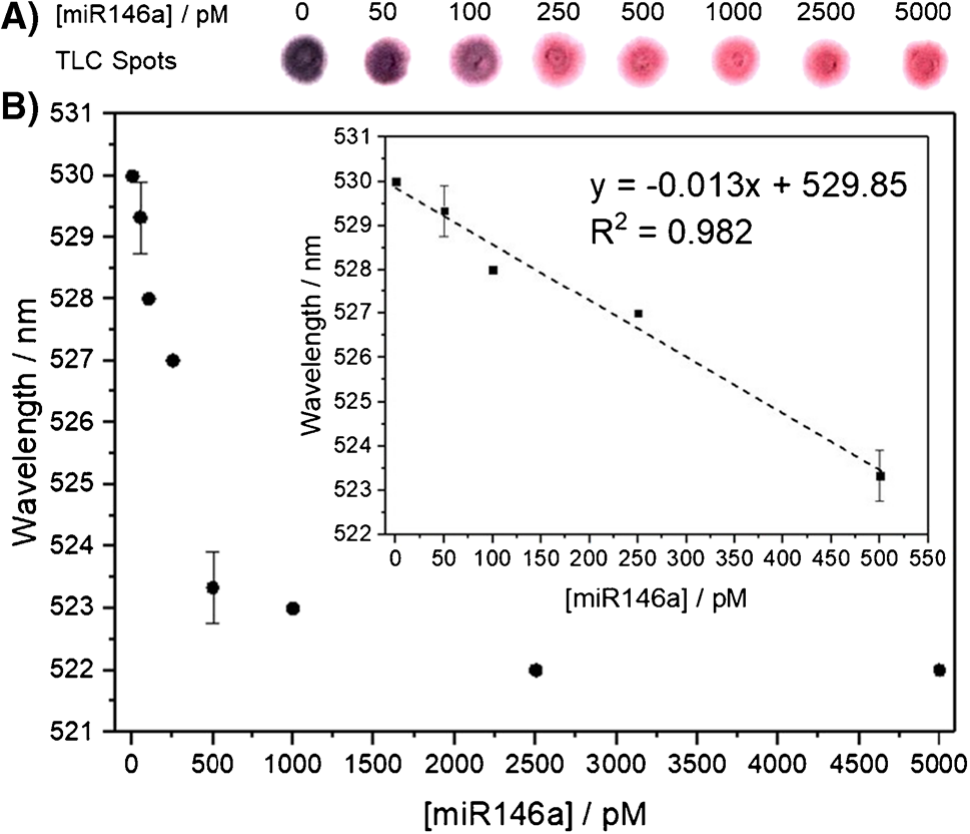
**A** Image of the AuNPs deposited onto the TLC plates in the presence of miR146a: For target concentrations higher than 100 pM, the color observed is pink, whereas for the blank, it is dark purple. **B** Representation of the variation in the SPR wavelength versus the miR146a concentration: 0 pM, 50 pM, 100 pM, 250 pM, 500 pM, 1000 pM, 2500 pM, and 5000 pM. All measurements were performed in quintuplicate (*n* = 5)

Evaluation of the analytical performance of the assay was carried out by analyzing the signal response obtained for the analysis of standards containing different target concentrations (0 pM, 50 pM, 100 pM, 250 pM, 500 pM, 1000 pM, 2500 pM, and 5000 pM) in quintuplicate (*n* = 5). This study was performed under optimized experimental conditions. The wavelength shifts of the SPR peak and the spots of the mixture deposited onto a TLC plate are displayed in Fig. 3. As can be observed in the image of the spots, the presence of miR146a at concentrations of 250 pM or higher produces a significant cleavage of the DNA-linker, showing a noticeable change in the spots from purple to pink. However, when targets were added with concentrations below 250 pM, the amount of DNA-linker cleaved by the MNAzyme was not sufficient to generate the pink color of the AuNPs when they were well dispersed in solution.

Although the purpose of this study was to elucidate the concentration level of miRNA that can be possible to be detected in a sample through a visual readout, spectroscopic measurements were also carried out. Figure 3A shows the spectra obtained for different target concentrations, in which a redshift toward higher wavelengths was observed when the concentration of the target was increased. Representation of the SPR wavelength versus different target concentrations shows a linear response at low concentrations of the target. The limit of detection, calculated as the concentration of the target that produces a change in the SPR wavelength equal to three times the standard deviation of the SPR wavelength of AuNPs in the absence of the target, is 12.7 pM. Notably, this LOD could be even lower by measuring the absorption at two wavelengths and with a ratiometric data treatment as reported previously [27]. In addition, besides the shift of the wavelength of the SPR peak, absorption values at a fixed wavelength could also be employed to carry out the detection of miR146a, as shown in Supplementary Fig. S7.

### Selectivity of the methodology for miR146a detection

**Fig. 4:**
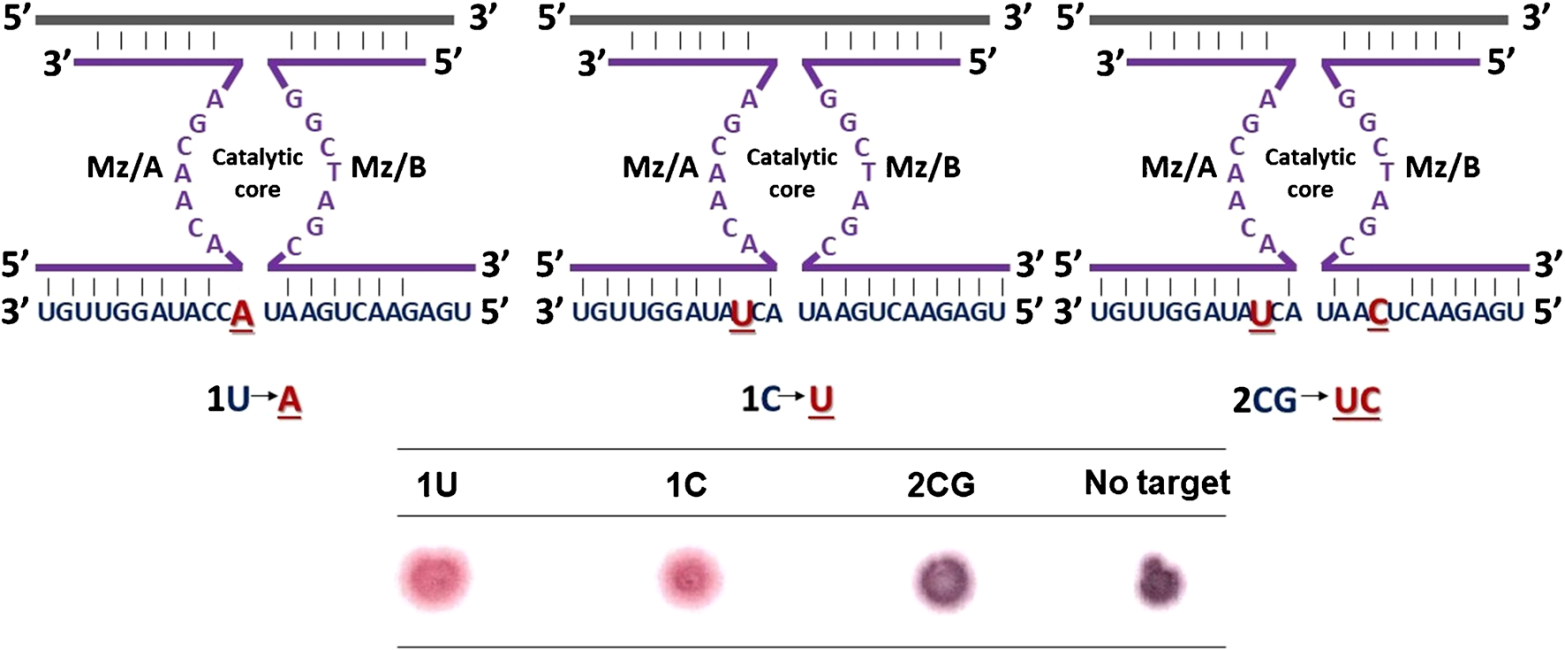
Detection of short RNA sequences after changing one and two bases (underlined in black) was changed from miR146a: modification of 1 base (1U and 1C) and two bases (2GC). These studies were carried out in triplicate (*n* = 3)

The selectivity of the proposed methodology toward the detection of miR146a was carried out by evaluating the analytical response achieved for similar miRNA sequences that only differ by 1 and 2 bases from miR146a. Figure 4 shows the variations evaluated, in which the sequence of the target is represented in red, and the modifications evaluated are underlined in black. The results obtained from experiments carried out in triplicate show that when a sample contains a sequence differing only in one base (e.g., 1U and 1C) from the target sequence, the MNAzyme becomes active, and the substrate is cleaved; thus, AuNPs remain in good colloidal stability, and the color observed is pink. These results indicate that it would not be possible to differentiate miR146a from another miRNA with the same sequence, but a single base mismatch is not possible. However, this issue would not constitute a drawback for this specific application. This is because miR146b, which differs from miR146a by one base mismatch, is another miRNA involved in mastitis infections.

Moreover, when the detection assay is carried out to analyze a sample containing a miRNA that exhibits a similar structure to miR146a but is modified with two bases (e.g., 2CG), the MNAzyme does not become active, and the cleavage of the target does not take place. Hence, aggregation of the AuNPs is observed, demonstrating the capability of the assay to differentiate miR146a from another microRNA with a two-base mismatch.

### Detection of miR146a in raw milk samples

The MNAzyme-based amplification strategy was evaluated for its ability to detect miR146a in raw milk samples. For this purpose, eight milk samples from healthy cows were obtained at the Regional Institute for Research and Agrofood Development of the Principality of Asturias (SERIDA) and spiked with known amounts of miR146a. These experiments were carried out in triplicate.

In milk samples, miR146a is predominantly found in the pellet formed after the sample is centrifuged to remove the fat and serum [28]. Hence, to evaluate the recovery of the spiked microRNA, milk samples were first centrifuged, the fat and serum top layers were discarded, and increasing amounts of miR146a were spiked into the pellet (0.025 pmol, 0.05 pmol, 0.125 pmol, 0.25 pmol, 0.5 pmol, 1.25 pmol, and 2.5 pmol). Afterward, the RNA extraction protocol was carried out using a commercial RNA extraction kit by following the instructions of the company. Once the RNA was extracted in 100 μL, the detection assay was carried out using optimal experimental conditions.

**Fig. 5:**
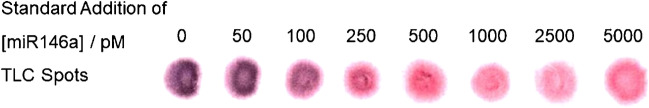
Image of the AuNPs deposited onto the TLC plates in the presence of miR146a in milk samples. Increasing miR146a standard additions of 0 pM, 50 pM, 100 pM, 250 pM, 500 pM, 1000 pM, 2500 pM, and 5000 pM were spiked into raw milk samples from healthy cows. At 250 pM of target concentration, the color observed is pink, while dark purple is observed for the blank. The standard additions and detection assays were carried out in triplicate (*n* = 3)

The results obtained from real samples are displayed in Fig. 5. As can be seen in the image of the spots, a clear positive result by means of visual detection is achieved for 250 pM miR146a with high repeatability. (Supplementary Fig. S8 shows the shift in wavelength of the SPR peak plotted against miR146a.) Assuming a 70% yield of RNA extraction, the concentration at which a change of color from purple to pink is undoubtedly observed is 250 pM. This is in agreement with the LOD obtained from the developed method. Hence, when the RNA extraction method was employed, no matrix effects were observed, making this method suitable for the detection of miR146a in real samples of raw milk.

To the best of our knowledge, the only study that characterizes the association between miRNA levels in milk and miRNA profile changes in mammary gland inflammation was performed by utilizing qPCR, a semiquantitative method [29]. Therefore, the methodology presented in this work could be employed to elucidate this association in quantitative terms.

## Conclusions

A genetic isothermal biosensor highly sensitive to miRNA visual detection was successfully developed. It is based on the synergy of a simple amplification scheme using MNAzymes. Then, AuNP aggregation is related to the presence of the target miRNA, producing a color change.

To the best of our knowledge, this is the first work that combines the use of MNAzymes and AuNPs for the detection of a short RNA sequence. The developed methodology has been successfully validated on raw milk samples obtained from healthy cows.

At present, few methods based on the colorimetric visual detection of miRNA have been reported (Table 1). The majority of those methods are based on nanoparticle aggregation assays using spectrophotometric measurements [31, 32]. The amplification schemes reported the use of protein enzymes [19, 29], which are temperature unstable and are typically based on complex and laborious methodologies, requiring successive temperature cycles. In the proposed method, after a previous RNA extraction from the sample is performed using a commercial kit capable of extracting the genetic material in approximately 30 min, the method is easily carried out instrumentation under isothermal conditions without complex instrumentation. This is a significant advantage to further develop an easy-to-use point-of-care device that sensitively detects miR146a in the lack of qualified personnel.

**Table 1:** Summary of the amplification strategies coupled to AuNPs based on aggregation assays published for miRNA detection

Method	Features	Application	LOD	Ref.
- Exponential amplification reaction (EXPAR)	- Spectrophotometry detection - Assay in 1 h - Enzyme dependent - Sample heating treatment	- miR-221-3p - Cell lysates from human cancer cell lines	46 fM	[30]
- Duplex-specific nuclease amplification (DSN)	- Spectrophotometry detection - Assay in 4 h and 30 min - Not successful for real samples	- miRNA let-7a - Not evaluated in real sample	80 pM	[20]
- Target catalyzed hairpin DNA assembling	- Spectrophotometry detection - Ratiometric measurements 630/520 nm - Assay in 4 h	- miRNA let-7a - RNA from human breast adenocarcinoma cells	3 pM	[31]
- Duplex-specific nuclease amplification	- Spectrophotometry detection - Ratiometric measurements 620/520 nm - Assay in 2 h	- miR-21 - Lysates from MCF-7 cells, HeLa cells, and L02 cells	300 pM	[32]
- Entropy-driven amplification (EDA) coupled with Nicking enzymes	- Spectrophotometry detection - Ratiometric measurements 530/650 nm - Assay in 2 h and 25 min - Requires temperature cycles	- miRNA let-7a - Diluted human serum samples	10 fM	[33]
- Exponential amplification reaction (EXPAR)	- Visual detection - Assay in 1 h - Enzyme dependent	- miRNA let-7a - Not evaluated in real sample	10 fM	[34]
- Multicomponent nucleic acid enzymes amplification (MNAzymes)	- Spectrophotometry detection - Visual detection - Assay in 1 h 20 min	- miR146a - Raw milk samples	250 pM/12 pM	This work

Briefly, visual detection of a target can be achieved through the reported assay without the need for additional instrumentation and qualified personnel. The method has been applied to the rapid, simple, low-cost, and highly sensitive detection of miRNA in raw milk samples. Moreover, it presents several advantages, as the method is easily adapted to detect other miRNAs by modifying the MNAzyme sequences. This technology has significant potential in many different fields in which the presence of miRNAs can be relevant, such as biomedical or environmental fields.

## Supplementary information

ESM 1 (DOCX 980 kb)
