# Supplementary material for: MNAzymes and gold nanoparticles as isothermal signal amplification strategy for visual detection of miRNA
Source: Mikrochim Acta. 2023 Jul 17;190(8):292. doi: 10.1007/s00604-023-05868-y (PMC10352400; doi:10.1007/s00604-023-05868-y)
Supplement: Supplementary file 1 — (DOCX 980 kb) [file 604_2023_5868_MOESM1_ESM.docx]

**ELECTRONIC SUPPORTING MATERIAL**

**MNAzymes and Gold Nanoparticles as Isothermal Signal Amplification Strategy for Visual Detection of miRNA**

Adrián Sánchez-Visedo^a^, Borja Gallego-Martínez^b^, Luis José Royo^c^, Ana Soldado^a^, Marta Valledor^d^, Juan Carlos Campo^d^, Francisco Javier Ferrero^d,^ *, José Manuel Costa-Fernández^a^, María Teresa Fernández-Argüelles^a,^*

^a^ Department of Physical and Analytical Chemistry, University of Oviedo, Avenida Julian Clavería 8, 33006 Oviedo (Asturias), Spain

^b^ Health Research Institute of Asturias, ISPA, Avenida Hospital Universitario s/n 33011 Oviedo (Asturias), Spain

^c^ Department of Functional Biology, Genetics, University of Oviedo, Avenida Julián Claveria, s/n 33006 Oviedo (Asturias), Spain

^d^ Department of Electrical, Electronic, Communications and Systems Engineering, University of Oviedo, Campus of Gijón, 33204, Gijón, Spain, University of Oviedo, Spain

***** Corresponding author: Maria Teresa Fernandez-Argüelles E-mail: [fernandezteresa@uniovi.es](mailto:fernandezteresa@uniovi.es)

Francisco Javier Ferrero, E-mail: [ferrero@uniovi.es](mailto:ferrero@uniovi.es)

**S.1. Surface functionalization of AuNPs**

Two different DNA sequences with a 5’-thiol-group (Probe 1) and 3’-thiol-group (Probe 2) were mixed with two colloidal dispersions of AuNPs, DNA_1_-AuNP and DNA_2_-AuNP, respectively. Probe 1 and Probe 2 present a chain of 10 adenines which acting as spacer between the thiol group and its corresponding sequence to ensure the appropriate orientation of the probes onto the NP surface. DNA_1_-AuNP and DNA_2_-AuNP were prepared as previously reported by mixing 20 µL of 100 nM AuNPs and 20 µL of 20 µM Probe 1 or Probe 2 (to obtain DNA_1_-AuNP and DNA_2_-AuNP, respectively) in ultrapure water with 0.01% v/v Tween-20, and 90 mM Trisodium Citrate HCl buffer, pH=3) for 30 min at room temperature [18].

To improve the colloidal stability of the AuNPs, and to block any DNA-free area on the NPs surface, after each corresponding thiolated DNA sequence was conjugated to the AuNPs, they were mixed with 10 µL of 2 mM thiolated methoxy polyethylene glycol (mPEG-SH_1000_) and incubated for 30 min at 60 ºC. Afterwards, to remove any excess DNA and mPEG-SH_1000_ from the supernatant, the DNA_1_-AuNP and DNA_2_-AuNP were purified by centrifuging the mixture three times at 8000 g and 4ºC for 30 minutes. Finally, both dispersions of the bioconjugated AuNPs were diluted with ultrapure water with 0.01% v/v Tween-20 to a final concentration of 5 nM, calculated using an extinction coefficient of 2.33 × 10^8^ M^−1^ cm^−1^ at λ = 521 nm for 15 nm AuNPs.

**S.2. Assesment of DNA:AuNPs functionalization and concentration**

Bioconjugation of thiolated RNA strands onto the surface of AuNPs has been carried out at different molar concentrations of DNA:AuNPs. To evaluate the efficiency of the bioconjugation, a gel electrophoresis using 1% agarose was run at 100 V for 40 minutes. Results obtained are displayed in Figure S1.


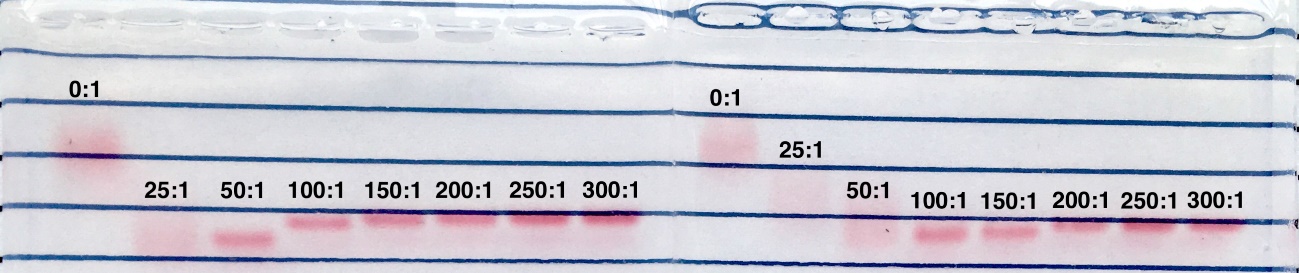


***Figure S1.*** *Evaluation of increasing DNA:AuNPs molar ratios in the bioconjugation step. The image on the left corresponds to Probe 1, and the image on the right to Probe 2. As the DNA:AuNPs molar ratio increases, the bioconjugate migrate slower due to an increase of the surface density of thiolated oligonucleotides onto the AuNPs. Results obtained, which present the same trend for Probe 1 and Probe 2, show that with a DNA:AuNPs molar ratio of 200:1, the surface of the AuNPs is already saturated with oligonucleotides. Hence, this bioconjugation molar ratio was selected for further experiments.*

DNA:AuNPs concentration was also evaluated to allow a visual detection of miR146a. AuNPs concentration was studied between 1 and 10 nM. Results obtained showed that very pale colors are observed in the TLC plate when the AuNPs concentration is 2.5 nM (see Figure S2). When the AuNPs is higher, the color observed is more intense, which allows distinguishing between aggregated and nonaggregated AuNPs with the naked eye. It is worth noting that, if the AuNPs is high, it will be necessary to have higher concentration of target to distinguish between aggregated and nonaggregated AuNPs. Hence, 5 nM concentration was selected for further experiments.


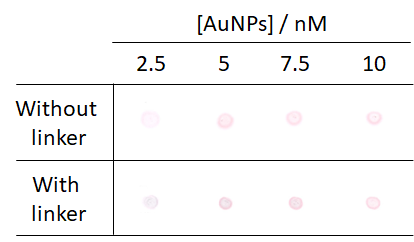


**Figure S2.** Images of the spots observed in the TLC plate for DNA:AuNPs at different concentrations, with linker (50 nM) and without linker

**S.3. Selection of experimental parameters affecting the amplification**

**S.3.1. Selection of the temperature of the assay**

The activity of the MNAzymes strongly depends on the temperature. It has been described that their cleavage activity is maximum at 50ºC, and rapidly decreases as the temperature is modified towards higher or lower values. Due to the changes in the sensor arms made in the MNAzyme made to detect a smaller sequence (i.e. miRNA), the effect of the temperature on the activity of the MNAzyme was evaluated.

Time-dependent catalytic signal was evaluated at different temperatures. For this purpose, absorption spectra in absence and presence of miR146a were recorded from 20 to 80 minutes at 40, 45, 50, 55 and 60ºC. In absence of target the MNAzymes are not active, so the DNA:linker is intact, and the AuNPs should be aggregated. In presence of a high concentration of miR146a (2500 pM), the linker will be cleaved by the MNAzyme, and as consequence, the AuNPs should not be aggregated. Results obtained are summarized in Figure S3, where the difference on the change of the wavelength of the SPR peak are displayed for the times and temperatures studied. As it can be observed, the difference on the wavelength of the SPR peak between presence and absence of target is maximum when the cleavage takes place at 50ºC for at least, 60 minutes. Higher or lower temperatures give rise to a smaller change, so the aforementioned conditions were selected for further experiments.

*
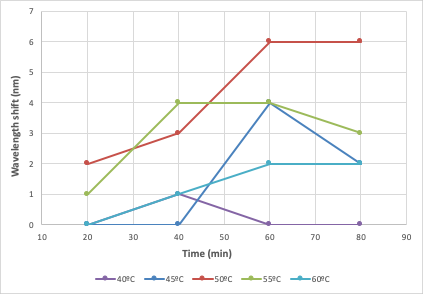
*

**Figure S3.** Time-dependent catalytic response at different temperatures. Wavelength shift was estimated as the difference between the SPR wavelength in absence of target minus the SPR wavelength in presence of 2500 pM miR146a. Wavelength shift was plotted against increasing times at 40, 45, 50, 55 and 60ºC. Results show that the maximum change is observed for incubation times of 60 minutes or higher at 50ºC.

In addition, the detection of different concentrations of miR146a was carried out at 40, 50 and 60ºC, respectively. Results obtained in our laboratory, which are displayed in Figure S4, are in agreement with those previously reported in the literature. As it can be observed, at 40 and 60ºC there is no noticeable shift on the wavelength of the SPR peak as the concentration of target increases. This fact suggests that there is not change in the aggregation state of the DNA:AuNPs associated to the presence of target. However, at 50ºC it can be observed that the SPR peak is located at 530 nm in absence of target, and at miR146a concentrations of 100 pM or higher, the SPR peak is shifted towards lower wavelengths. These results suggest that cleavage of the linker due to the activity of the MNAzyme in presence of the target only takes place at 50ºC. Hence, the effect of the catalytic time-dependent activity was evaluated at 50ºC


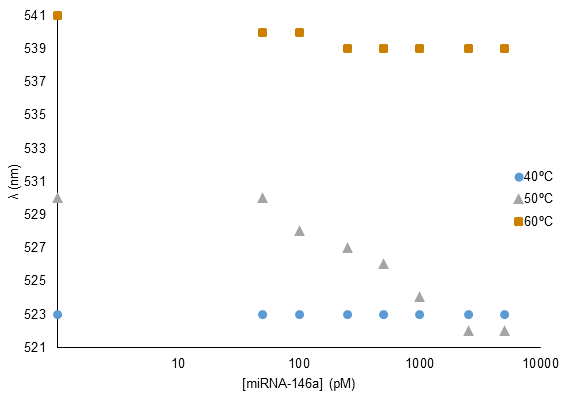


**Figure S4.** SPR wavelength shift of DNA-AuNPs evaluated at different temperatures, in presence of different miR146a concentrations of 0 pM, 50 pM, 100 pM, 250 pM, 500 pM, 1000 pM, 2500 pM, 5000. As it can be observed, at 40ºC and 60ºC there is not noticeable shift on the wavelength of the SPR peak as the target concentration increases. At 50ºC a shift on the wavelength can be noticed already in presence of miR146a 100 pM.

**S.3.2. Evaluation of incubation times**

The assay is carried out in two stages: a first step where the cleavage of the DNA-linker takes place, and a second step where hybridization of the DNA:AuNPs with the intact or cleaved DNA-linker occurs. Therefore, incubation time of both steps has been studied.

In order to evaluate the effect of time in the catalytic activity of the MNAzymes, detection of different concentrations of target was carried out at 50ºC for 20, 40, 60 and 70 minutes for the first step (while the time of the second step remained constant). A comparison of the results can be observed in Figure S5-E. As shown in Figure S5-A and S5-B, for incubation times of 20 and 40 minutes, the change in the wavelength of the SPR as consequence of variations in the aggregation state of the AuNPs is only observed at target concentrations above 250 nM (and with relatively big error bars). This indicates that in such short period of time, MNAzymes are capable of cleaving enough DNA:linker in presence of high concentrations of target. However, when the incubation takes place during 60 or 70 minutes (Figure S5-C and S5-D, respectively), similar and more reproducible changes can be observed at target concentrations of 100 nM (smaller error bars when compared to incubation times of 20 and 40 minutes, respectively). Therefore, incubation time of 60 minutes for the first step was selected to further experiments.”

**Figure S5.** SPR wavelength shift of DNA-AuNPs evaluated in presence of different miR146a concentrations of 0 pM, 50 pM, 100 pM, 250 pM, 500 pM, 1000 pM, 2500 pM, 5000, incubated at 50ºC for 20, 40, 60 and 70 minutes in the first cleavage stage. Graphs A to D are the results obtained for the different incubation times plotted with error bars, and Figure 5S-E shows a comparison of the results without the error bars.

During the second step of incubation, DNA:AuNPs are added to the mixture. Hybridization of the bioconjugates with the DNA:linker takes place, and aggregation of the AuNPs occurs if the linker is intact (i.e.: in absence of target), whereas the AuNPs are well dispersed in the linker has been cleaved by the MNAzyme in presence of miR146a during the first step. Results obtained from this study are shown in Figure S6. Graphs S6-A to S6-E show the results obtained for each incubation time individually with the error bars. Graph S6-F shows a comparison of the results obtained. As it can be observed, when the incubation time is low (e.g.: 10-15 minutes), it is not possible to observe a change in the SPR peak for low concentrations of miR146a. However, when the incubation time is of 20-25 or 40 minutes, a noticeable change in the SPR peak can be observed for 100 pM miR146a. Therefore, in order to carry out the assay within the shortest possible time, an incubation time of 20 minutes was selected for the second step.


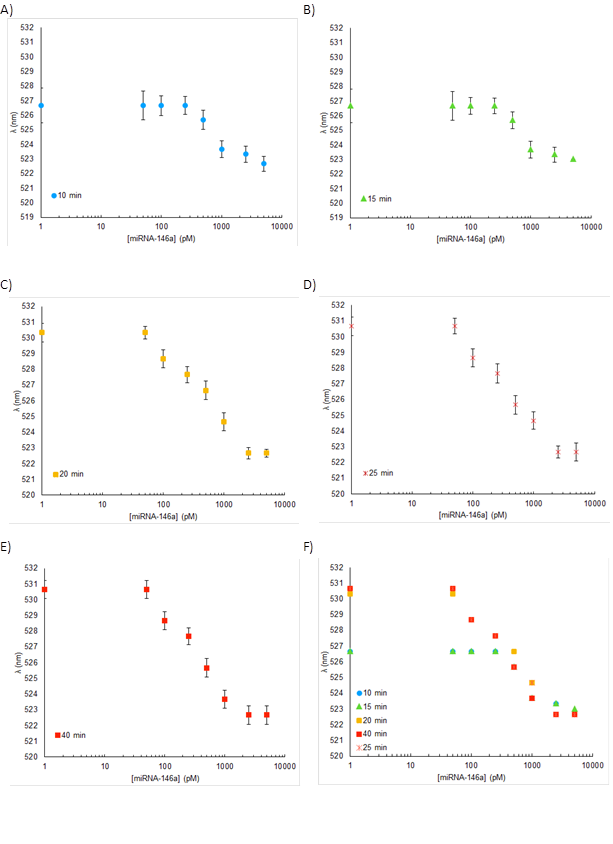


**Figure S6.** SPR wavelength shift of DNA-AuNPs evaluated in presence of different miR146a concentrations of 0 pM, 50 pM, 100 pM, 250 pM, 500 pM, 1000 pM, 2500 pM, 5000, incubated at 50ºC for 60 min in the first step, and 10, 15, 20, 25 and 40 minutes in the second aggregation step. Graphs A to E show results plotted with error bars, and Figure 6S-F shows a comparison of the results at different incubation times without error bars.

**S.4. Absorption measurements in presence of increasing concentrations of miR146a**

Despite the assay developed in this work aims to carry out a visual detection of miR146a, absorption measurements at a constant wavelength in presence of different concentrations of the target were also performed. As can be observed in Figure S7, when the absorbance is measured at 522 nm, the trend observed is that the absorbance decreases linearly between 0 and 2500 pM. This would allow, together with measurements of the shift of the wavelength, to carry out quantification of miR146 if required for the application.


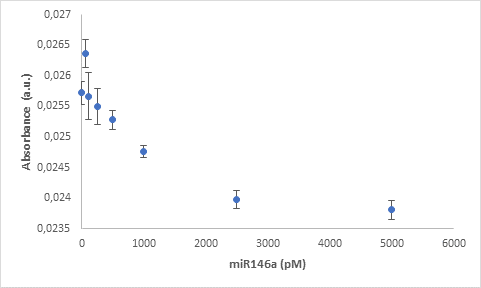


**Figure S7.** Absorbance measured at 522 nm at different miR146a concentrations of 0 pM, 50 pM, 100 pM, 250 pM, 500 pM, 1000 pM, 2500 pM, 5000 pM. Measurements were carried out in triplicate (n=3)

**S.5. Detection of miR145a in raw milk samples**

Detection of miR146a in raw milk samples was carried out in both using two visual readouts: visual detection and by measuring the shift of the wavelength of the SPR peak. Results are summarized in Figure S8. It can be noticed that for a visual readout, a250 pM concentration of miR146a gives rise to a noticeable change of the colour of the spots in the TLC plate. This would allow to give rise to a qualitative response, because no difference can be noticed for higher concentrations. However, in case a quantification of the miR146a is required, the concentration can be estimated by plotting the shift of the wavelength of the SPR peak at different concentrations of target. This would allow to provide an estimation of the concentration of target, despite this is not the aim of this work.


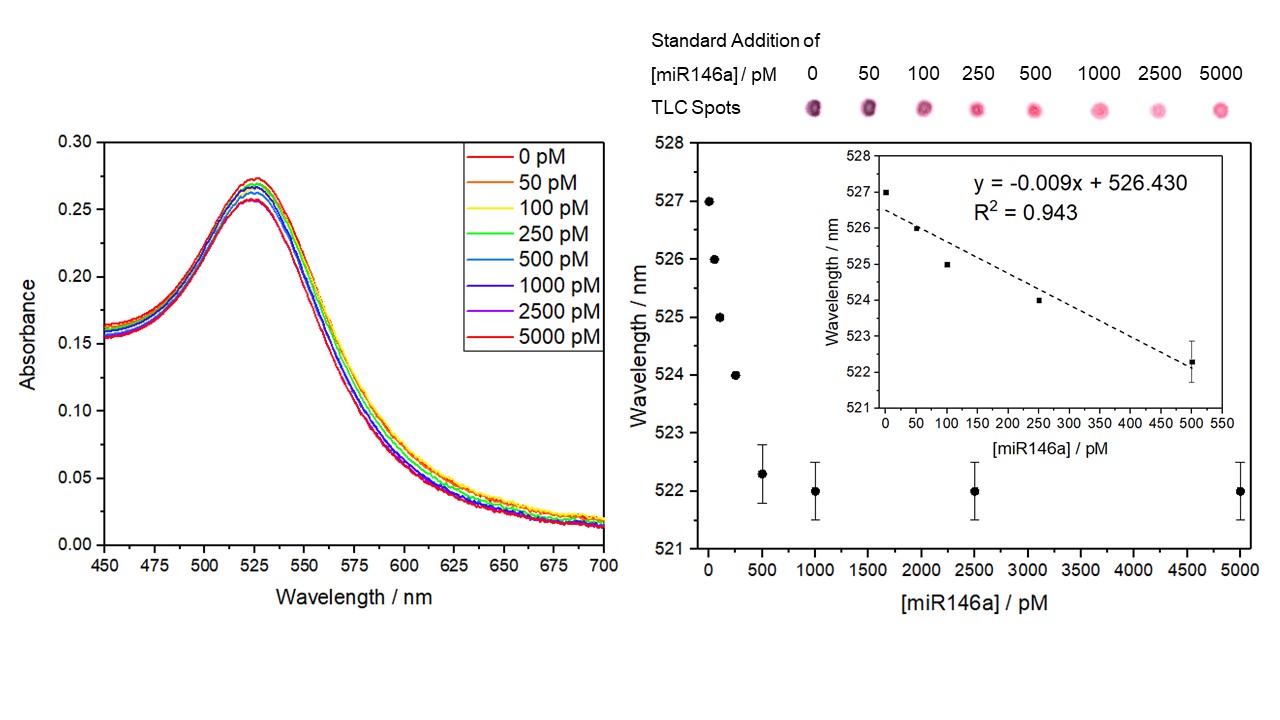


**Figure S8.** SPR wavelength and TLC Spots of AuNPs at different miR146a standard additions of 0 pM, 50 pM, 100 pM, 250 pM, 500 pM, 1000 pM, 2500 pM, 5000 pM to milk samples. Both, the spectroscopic measurements and TLC spots were carried out in triplicate (n=3)
